# Cannabis and cannabinoid use in autism spectrum disorder: a systematic review

**DOI:** 10.47626/2237-6089-2020-0149

**Published:** 2021-05-08

**Authors:** Estácio Amaro da Silva, Wandersonia Moreira Brito Medeiros, Nelson Torro, João Marçal Medeiros de Sousa, Igor Bronzeado Cahino Moura de Almeida, Filipe Barbosa da Costa, Katiúscia Moreira Pontes, Eliane Lima Guerra Nunes, Marine Diniz da Rosa, Katy Lísias Gondim Dias de Albuquerque

**Affiliations:** 1 Departamento de Psicologia Universidade Federal da Paraíba João Pessoa PB Brazil Departamento de Psicologia, Universidade Federal da Paraíba (UFPB), João Pessoa, PB, Brazil.; 2 UFPB João Pessoa PB Brazil UFPB, João Pessoa, PB, Brazil.; 3 Sociedade Brasileira de Estudo da Cannabis Sativa Sociedade Brasileira de Estudo da Cannabis Sativa.; 4 Departamento de Fonoaudiologia UFPB João Pessoa PB Brazil Departamento de Fonoaudiologia, UFPB, João Pessoa, PB, Brazil.; 5 Departamento de Fisiologia e Patologia UFPB João Pessoa, PB Brazil Departamento de Fisiologia e Patologia, UFPB, João Pessoa, PB, Brazil.

**Keywords:** Cannabis, cannabidiol, cannabinoid, autism, systematic review

## Abstract

**Introduction:**

Autism spectrum disorder (ASD) is a neurodevelopmental disorder characterized by persistent deficits in social communication and social interaction, associated with the presence of restricted and repetitive patterns of behavior, interests, or activities. Cannabis has been used to alleviate symptoms associated with ASD.

**Method:**

We carried out a systematic review of studies that investigated the clinical effects of cannabis and cannabinoid use on ASD, according to the Preferred Reporting Items for Systematic Reviews and Meta-Analyses (PRISMA checklist). The search was carried out in four databases: MEDLINE/PubMed, Scientific Electronic Library Online (SciELO), Scopus, and Web of Science. No limits were established for language during the selection process. Nine studies were selected and analyzed.

**Results:**

Some studies showed that cannabis products reduced the number and/or intensity of different symptoms, including hyperactivity, attacks of self-mutilation and anger, sleep problems, anxiety, restlessness, psychomotor agitation, irritability, aggressiveness perseverance, and depression. Moreover, they found an improvement in cognition, sensory sensitivity, attention, social interaction, and language. The most common adverse effects were sleep disorders, restlessness, nervousness and change in appetite.

**Conclusion:**

Cannabis and cannabinoids may have promising effects in the treatment of symptoms related to ASD, and can be used as a therapeutic alternative in the relief of those symptoms. However, randomized, blind, placebo-controlled clinical trials are necessary to clarify findings on the effects of cannabis and its cannabinoids in individuals with ASD.

**Systematic review registration:**

International Prospective Register of Systematic Reviews (PROSPERO), code 164161.

## Introduction

Autism spectrum disorder (ASD) is a neurodevelopmental disorder characterized by persistent deficits in social communication and social interaction, in multiple contexts, associated with the presence of restricted and repetitive patterns of behavior, interests, or activities.^1^ In a multicenter epidemiological study performed in 2012, involving nine countries, the estimated average prevalence of ASD was 62 individuals per 10,000 inhabitants.^2^ Children with autism commonly exhibit comorbidities such as hyperactivity, self-harm, aggression, restlessness, anxiety and sleep disorders.^3^ This type of behavior favors social exclusion and limits the child’s abilities, causing more distress to caregivers.^4^

Conventional medical treatment includes several psychotropic drugs such as atypical antipsychotics, selective serotonin reuptake inhibitors, stimulants and anxiolytics; they do not treat ASD, but aim to eliminate inappropriate behavior, such as psychomotor agitation, aggressiveness, and obsessive-compulsive symptoms.^5-8^ They may lead to severe side effects such as nephropathy, hepatopathy, and metabolic syndromes, among others.^9^ Unfortunately, 40% of children with autism and disruptive behaviors do not respond well to standard medical and behavioral treatment.^4^ This carries a high cost for the individual and society, causing life expectancy to be reduced by 20 years in patients with autism compared to the population average.^10^

Among the possible pharmacological treatments, researchers began to explore other therapeutic alternatives, such as the use of substances derived from *Cannabis sativa.*^11^ Cannabidiol (CBD) represents one of the major components of the plant, having been studied in several disorders. At present, preliminary evidence suggests that CBD can relieve spasticity,^12^ pain, sleep disorders,^13^ improve mobility in multiple sclerosis,^14^ in addition to relieving anxious symptoms and social phobia^15^; however, further studies are needed to prove its effectiveness.

In autism, cannabis and cannabinoids have also been used to treat symptomatic conditions.^16,17^ CBD, and some other compounds in the plant, interact with the endocannabinoid system and can modulate different aspects related to cognition, socioemotional responses, susceptibility to seizures, nociception and neuronal plasticity, which are often altered in autism.^18-21^ In mammals, the endocannabinoid system is mainly composed of two receptors, CB1 and CB2, endocannabinoids (endogenous substances that activate CB1 and CB2 receptors) and the enzymes responsible for their synthesis and metabolism.^22^

CB1 receptors are expressed in both the central and peripheral nervous systems, with their most abundant expression in basal ganglia nuclei and pre-synaptic GABAergic and glutamatergic neurons.^23^ Considering that the endocannabinoid system modulates emotional responses, mood, behavioral reactions to the context and social interaction, investigators have started to formulate the hypothesis that changes in this system would be present in the autistic phenotype.^24^ Aran et al.^25^ observed reduced levels of endocannabinoids, such as anandamide (AEA), palmitoylethanolamide (PEA) and oleoethanolamine (OEA), in plasma samples from 93 children with ASD, suggesting the use of such substances as possible biomarkers for diagnosis. Pretzsch et al.^26^ reported that CBD can change the levels of the metabolites Glx (glutamate + glutamine) and gamma-aminobutyric acid (GABA) – the metabolites that contribute to the regulation of excitatory and inhibitory neurotransmission, both in typical development and in ASD. In an uncontrolled single-case study, delta-9 tetra-hydrocannabinol (∆9-THC) was administered to a 6-year-old autistic boy who was not taking any medication for 6 months. After the treatment period, there was a decrease in the scores of hyperactivity, lethargy, stereotyped behavior and language change, leading the authors to suggest the use of the substance as a resource to other treatments and early interventions.^27^

Thus, evidence has indicated that *Cannabis sativa* derivatives can alleviate symptoms associated with ASD, although there is still no consistent evidence about its efficacy, safety and tolerability, since no randomized, double-blind, placebo-controlled clinical trial with cannabis and cannabinoid for the treatment of the core symptoms of autism and coexisting symptoms have been conducted to date (only prospective studies are currently available). The research so far performed has shown that there are few side effects and, when they do occur, they are generally mild/moderate and transitory. In order to analyze such aspects, we carried out a systematic review of studies that used cannabis derivatives in autism, considering the evolution of symptoms and clinical improvement of these individuals.

## Method

In October 2020, we carried out a systematic literature review following the rules of the Preferred Reporting Items for Systematic Reviews and Meta-Analyses (PRISMA) system. The study was registered in the International Prospective Register of Systematic Reviews database (PROSPERO) with the code 164161.

To support the review, the following questions were asked: 1) What is the efficacy, safety and tolerability of cannabis and cannabinoids in treating symptoms of ASD? 2) What are the main instruments used to assess the evolution of symptoms and clinical improvement?

**Figure 1:**
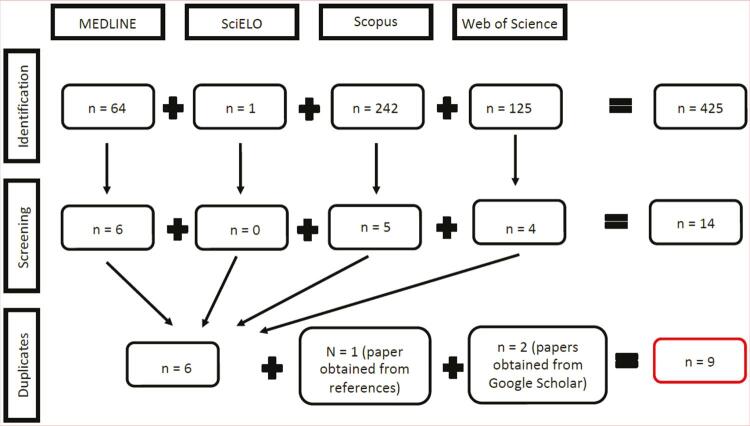
Study selection flowchart according to the Preferred Reporting Items for Systematic Reviews and Meta-Analyses (PRISMA) for cannabis and cannabinoid use in autism spectrum disorder.

The search was carried out in four databases: MEDLINE/PubMed, Scientific Electronic Library Online (SciELO), Scopus, and Web of Science. Additional studies were retrieved by checking the references of the selected articles. Finally, a search was performed using the Google Scholar tool. The search strategy for the databases was defined based on terms found in the title or abstract, using descriptors related to cannabis (cannabis, cannabidiol, cannabinoid, CBD, marijuana, marihuana, and hemp) and also descriptors related to autism (autistic, autism, Asperger, and pervasive development disorder). During the selection process, no restrictions were applied in terms of language, e,g., any article found was included in the eligibility analysis. Descriptors were included in quotation marks, and the search operators “AND” and “OR” were used. Cannabis-related terms were grouped using the “OR” operator; terms related to autism were grouped similarly. Then, these two groups of related terms were added and joined by the “AND” operator (Figure 1).

We included all articles published until October 2020, in any language, in the form of clinical trials or case studies involving human beings. Articles unrelated to the topic, i.e., those reporting on illicit or recreational use of cannabis, as well as abstracts, book chapters, animal studies, and research on other pathologies or changes that were associated with signs and symptoms similar to those observed in autism, were rejected.

The articles found in the databases were initially screened by reading their titles and abstracts. Subsequently, those articles considered to meet the proposed topic were read in full. At the end of the screening phase, we browsed the references of the articles ultimately selected in search of other studies that met the eligibility criteria.

The search and screening of the selected articles were carried out simultaneously and independently by two authors. In the end, the disagreements found were sent to another author, to make the final decision about whether or not to include a certain study, but always checking the eligibility criteria.

The searches conducted in the MEDLINE/PubMed, SciELO, Scopus, and Web of Science databases yielded 64, 1, 242, and 125 articles, respectively. Of these, respectively, 58, 1, 237, and 121 articles were eliminated because they did not meet the inclusion criteria. Thus, 14 studies were found, which, after eliminating duplicates, resulted in six articles. From the browsing of references of these six studies, another paper was selected to be part of the review, making a total of seven selected articles. Finally, the search carried out on Google Scholar yielded two more studies, reaching a final total of nine articles included in this systematic review, in accordance with the inclusion and exclusion criteria adopted (Figure 1).

The data extraction method of each study consisted in filling a standardized information sheet. One reviewer extracted the scientific data, and a second reviewer verified the acquired information. Disagreements were resolved by discussion and consensus among the authors-reviewers.

## Results

The initial results of the search returned 425 articles. After the first screening, 411 were excluded for not meeting the inclusion or exclusion criteria, due to the following reasons: primary research on the endocannabinoid system or on other mental disorders (172), book chapters, conferences or editorials (87), research on animals (26), review articles (85), research investigating the effects of illicit or recreative use of cannabis (35), studies on other disorders and conditions that overlap with some symptoms of ASD (6). Afterwards, eight articles were found to be duplicates and were therefore excluded, resulting in six articles retrieved from MEDLINE/PubMed, Scopus, and Web of Science. Finally, there was the addition of two studies found in Google Scholar and one article found through the analysis of references of the previously selected articles, at a final total of nine articles for analysis (Table 1).

**Table 1:** Studies selected for systematic review of the use of cannabis and cannabinoids for ASD

Title, authors and year	Country	Sample	Method	Results	Conclusion
Brief report: Cannabidiol rich cannabis in children with autism spectrum disorder and severe behavioral problems--a retrospective feasibility study Aran et al., 2018^17^	Israel	60 children (mean 11.8 years old, SD 3.5 years), 83% male, 77% with low cognitive function (according to ADOS or CARS), all with severe behavior problems (6 or 7, according to CGI-S).	Retrospective analysis of autistic children with behavioral changes refractory to conventional treatment at the Shaare Zedek Medical Center (Jerusalem, Israel), with medical prescription of cannabis for 7 to 13 months with plant extract containing CBD and THC at 20:1 (in poorly responsive cases, 6:1).	The average daily dose was 3.8±2.6 mg/kg/day of CBD and 0.29±0.22 mg/kg/day of THC for the 44 children who received three doses/day. For the 16 who received two doses, 1.8±1.6 mg/kg/day of CBD and 0.22±0.14 mg/kg/day of THC was the average dose received. 51% presented one side effect, the most common being: sleep disorders (14%), restlessness (9%), nervousness (9%), and loss of appetite (9%). In the HSQ score, 29% had an average improvement of 1.38±1.79 (median = 0.81). In the APSI score, it was 0.66±0.74 (median = 0.53).	There was a significant improvement in behavioral problems that was reported in 61% of children, in the CGIC; in anxiety: 39% and in communication problems: 47%. High concentration of THC (6:1-CBD) can lead to a psychotic episode.
Oral cannabidiol use in children with autism spectrum disorder to treat related symptoms and comorbidities Barchel et al., 2018^16^	Israel	53 children (mean 11 years of age, SD 4 to 22 years) received CBD for an average of 66 days (SD 30-588 days).	Administration of oil with CBD and THC (20:1), orally, with telephone interviews conducted every two weeks with parents or caregivers, asking about changes in symptoms, the data obtained were analyzed independently by specialists in search of these changes in symptoms and safety of medicines. The improvement resulting from CBD was also compared with conventional treatment for ASD.	Self-harm and anger bouts (n = 34) improved in 67.6% and worsened in 8.8% of the participants. Symptoms of hyperactivity (n = 38) improved in 68.4%, did not change in 28.9%, and worsened in 2.6% of the subjects. Sleep problems (n = 21) improved in 71.4% and worsened in 4.7%. Anxiety (n = 17) improved in 47.1% and worsened in 23.5% of the participants. Adverse effects, mostly somnolence and change in appetite, were mild.	A comparison of symptom improvement between CBD treatment and conventional treatment was analyzed using the binomial test. Parents’ reports suggest that CBD may improve symptoms related to ASD.
Effects of cannabidiol on brain excitation and inhibition systems: a randomized placebo-controlled single dose trial during magnetic resonance spectroscopy in adults with and without autism spectrum disorder Pretzsch et al., 2019^26^	England	34 people (neurotypical control n = 17, ASD n = 17). All with IQ greater than 70.	Patients were allocated in a randomized order: about half in each group participated in the placebo before CBD (600 mg oral solution) and the other half participated in CBD before the placebo. After administration, placebo or CBD, a check was scheduled to coincide with the maximum plasma concentration (2 h). It was evaluated by magnetic resonance spectroscopic imaging.	It was seen that patients with ASD had a drop in IQ compared to neurotypical controls (F_1_ = 5,781; p = 0.022), but the difference in IQ did not influence the results: ASD (r < -0.008; p > 0.698); neurotypical (r < 0.068; p > 0.235). The excitatory mechanisms of response to glutamate were comparable, regardless of diagnosis, however the inhibitory response by GABA + was altered in ASD. There was a difference in the results found in the images in relation to the placebo group.	CBD can change the levels of glutamate, glutamine and GABA +, regulators of excitatory and inhibitory neurotransmission. The autistic brain reacts differently to GABA+, which helps to understand the mechanisms and targets of treatment for ASD.
The effect of cannabidiol (CBD) on low-frequency activity and functional connectivity in the brain of adults with and without autism spectrum disorder (ASD) Pretzsch et al., 2019^28^	England	34 people (17 with ASD and 17 without).	CBD 600 mg oral solution. Functional magnetic resonance imaging was used for evaluation.	CBD is able to alter the fALFF in the cerebellar vermis, center of perception of gravity, right fusiform gyrus. The connectivity function (FC) in the vermis increased in the left and right caudal portion, however it reduced between the vermis and the occipital temporal part of the left middle temporal gyrus, the right supramarginal anterior gyrus, the left upper parietal lobe and the gyrus upper left front; none of these effects were observed significantly in the brains of healthy people. There was a difference in the results found in the images in relation to the placebo group.	First evidence of neuromodulation made from the administration of CBD in fALFF and FC in the brains of adults with autism. CBD was able to alter crucial properties of brain function in key areas that are altered in ASD.
Real life experience of medical cannabis treatment in autism: analysis of safety and efficacy Bar-Lev Schleider et al., 2019^29^	Israel	188 patients with ASD with a mean age of 12 years, SD ± 7 years, younger than 5 years (14). 81.9% of the male gender.	Cannabis oil enriched with 30% CBD and 1.5% THC (3 times a day, sublingual) was used, oil enriched by an average of 61.5 + -79.5 mg CBD and 3 + -4 mg THC. The team initially and periodically evaluated the health status, assessed the medical history and administered medical questionnaires.	In 6 months (49.5% of sample loss), 91% of cases of restlessness improved; 90.3% of anger bouts; 85.2% of agitation; 78.1% problems with sleep; among other symptoms. There was at least one side effect in 25.2%, which were: restlessness (6.6%), drowsiness (3.2%), psychoactive effect (3.2%), increased appetite (3.2%), digestive problems (3.2%), dry mouth (2.2%) and lack of appetite (2.2%).	The use of cannabis for ASD is well tolerated, safe and appears to be effective in relieving symptoms (especially seizures, depression, restlessness and bouts of anger). There was great acceptability of the treatment, with only less than 15% of dropouts in a 6-month follow-up. More than 80% of parents reported a significant global improvement in children.
Use of dronabinol (delta-9-THC) in autism: A prospective single-case-study with an early infantile autistic child Kurz & Blaas, 2010^27^	Austria	Boy, 6 years old, (diagnosed at 3) via DSM-IV criteria and confirmed by ADOS and ADI.	Drops of dronabinol dissolved in sesame oil, with one drop initially (0.62 mg) in the morning up to the maximum dose of 2 drops in the morning, with a total daily dose of 3.62 mg of dronabinol. 6-month follow-up (without adding other new therapies or changing existing care measures). Symptom severity was assessed using the ABC questionnaire.	Hyperactivity decreased by 27 points, lethargy reduced by 25 points, irritability decreased by 12 points, stereotypy reduced by 7 points and inappropriate speech decreased by 6 points in six months.	This isolated case suggests that dronabinol may reduce the symptoms of autism in children, perhaps by modifying cannabinoid levels in the central nervous system.
Rating of the safety and effectiveness of marijuana, THC/CBD, and CBD for autism spectrum disorders: results of two national surveys Adams et al., 2019^30^	United States	156 participants who already used cannabis in its derived forms.	The National Survey on Treatment Effectiveness for Autism (NSTEA) started collecting data in 2017 and continues to collect online. Marijuana is studied in the following forms: flower, edible, vaporized, gums, tincture, leaf and other forms; THC/CBD combination in the following forms: oil, gums, edible, tincture, vaporized and all methods; and only CBD in the following forms: oil, tincture, gums and others.	Reported improvements: calm (58-71%); irritability (46-65%); aggression/agitation (43-58%); sleep (30-58%); drowsiness (32-46%); hyperactivity (26-39%); sensory sensitivity (28-32%); cognition (32-46%); attention (26-42%); social interaction (26-42%); language (26-38%); perseverance (22-27%); depression (16-41%). Adverse effects of CBD, uncommon: behavioral problems (5%), decreased cognition (4%), fatigue (4%), aggression/agitation (4%). All these side effects were mild and/or transient.	The primary reported benefits were calming effects, including improved anxiety, irritability, aggression/agitation, hyperactivity, and sleep. There were also improvements in the symptoms of ASD. There were few adverse effects for THC/CBD and CBD and mild for marijuana.
Effects of CBD-enriched *Cannabis sativa* extract on autism spectrum disorder symptoms: an observational study of 18 participants undergoing compassionate use Fleury-Teixeira et al., 2019^31^	Brazil	18 patients: 11 without a history of epilepsy, 2 with a previous history of epilepsy but without seizures for over a year, and 5 with epilepsy and still with seizures.	Extract enriched with CBD in the ratio CBD/THC 75:1. Average of 4.6 mg/kg/day of CBD and 0.06 mg/kg/day of THC. The individual doses were based on previous studies with patients with refractory epilepsy associated with autism. The average initial dose was 2.9 mg/kg/day and dose adjustments were made throughout the treatment.	80% of patients improved in more than 30% of the three items assessed: sleep disorders, epileptic seizures, and behavioral changes. In addition, signs of improvement were reported for motor development; communication and interaction; and cognitive performance. The adverse effects were: moderate drowsiness and irritability (three cases each), diarrhea, increased appetite, conjunctival hyperemia, and increased body temperature (one case each). All these side effects were mild and/or transient.	Several therapeutic benefits of the CBD-enriched preparation that extends to ASD symptoms have been noted, even in non-epileptic patients. This study pointed to a potential risk of paradoxical effects when introducing cannabinoids to a patient using a combination of drugs that include antipsychotics. This highlights the need for extra vigilance and a gradual increase in the dosage of cannabinoids in patients receiving many medications.
Effects of cannabidivarin (CBDV) on brain excitation and inhibition systems in adults with and without autism spectrum disorder (ASD): a single dose trial during magnetic resonance spectroscopy Pretzsch et al., 2019^32^	England	34 participants, around 28.47 (6.55) years old in the control group and 31.29 (9.94) in those with ASD, among them 17 people, diagnosed by ICD-10, with severe symptoms evaluated by ADOS and ADI.	Randomized, double-blind, crossover study using magnetic resonance spectroscopic imaging comparing glutamate and GABA levels after the use of placebo and 600 mg CBDV. Information was collected from the dorsomedial region of the prefrontal cortex and the left basal ganglia (areas related to ASD) after 2 h (plasma peak of the substance) of administration.	Tests performed at least 13 days after using the drug/placebo indicated that CBDV increased the levels of glutamate in the left basal ganglia in both groups, but in those with ASD despite this increase, the basal concentration of the substance decreased. CBDV did not alter the levels of glutamate or GABA in the medial dorsal region of the prefrontal cortex of either group. There was a difference in the results found in the images in relation to the placebo group.	CBDV modulates the levels of glutamine/GABA in the left basal ganglia, with individual variations depending on the biochemistry of the individual base (CBDV increased the levels of glutamate in autistic low baseline amounts, opposite to those who already had it high in baseline). Future studies should evaluate the effect of CBDV on behavior and whether the response to an acute dose can predict therapeutic success in patients with ASD.

ABC = Aberrant Behavior Checklist; ADI = Autism Diagnostic Interview; ADOS = Autism Diagnostic Observation Schedule; APSI = Autism Parenting Stress Index; ASD = autism spectrum disorder; CARS = Childhood Autism Rating Scale; CBD = cannabidiol; CBDV = cannabidivarin; CGIC = Caregiver Global Impression of Change; CGI-S = Clinical Global Impression Scale – Severity; DSM-IV = Diagnostic and Statistical Manual of Mental Disorders, 4th edition; fALFF = fractional amplitude of low-frequency fluctuations; FC = connectivity function; GABA = gamma-aminobutyric acid; HSQ = Home Situations Questionnaire; ICD-10 = International Classification of Diseases, 10th revision; IQ = intelligence quotient; SD = standard deviation; TBI = craniocerebral trauma; THC = tetrahydrocannabinol.

The countries of origin of the studies included in the systematic review were: Israel (three studies), England (three studies), Brazil (one study), Austria (one study), and the United States (one study).

Five studies used cannabis extract, in the presentation of CBD-rich oil,^16,17,28,30,31^ two studies used CBD in oral solution,^26,28^ one study used dronabinol, which is a synthetic analogue of THC (tetrahydrocannabinol), dissolved in sesame oil,^27^ and one study used cannabidivarin (CBDV)^32^ (Table 1).

The studies using CBD-enriched cannabis oil showed a variation between the proportions of CBD and THC, ranging from 6 to 75% CBD combined with 1 to 1.5% THC. Those who used pure CBD used a dose of 600 mg (oral solution), dronabinol was used at a dose ranging between of 0.62 and 3.62 mg/day (dissolved in sesame oil), and cannabidivarin was used at a dose of 600 mg.

The samples were composed of: 1) children in three studies^16,17,27^; 2) children and adolescents in one study, with ages ranging from 5 to 19 years^29^; and 3) adults in three studies.^26,28,32^ Two studies did not specify the age group.^30,31^

Only three studies used any imaging exam, namely, magnetic resonance spectroscopic imaging after the intervention with CBD in two studies and with CBDV in one study to search for brain changes.^26,28,32^ The other studies used questionnaires, forms and subjective reports of family members or caregivers. Of the nine studies selected for the systematic review, with the age groups already described above, one had placebo allocated to participants in a randomized order, with half of the sample using CBD before vs. half after using the placebo^26^; two were randomized, double-blind and placebo-controlled^28,32^; in the remaining studies, the intervention of cannabis or cannabinoids was administered without randomization, as previously described. It is important to note that none of the studies evaluated included the cognitive assessment of children through neuropsychological tests.

Regarding the results found, the studies that tested cannabis to improve behavior showed improvement in many individuals with ASD. The following symptoms were targeted: bouts of self-mutilation and anger, hyperactivity, sleep problems, anxiety, restlessness, psychomotor agitation, irritability, aggressiveness, sensory sensitivity, cognition, attention, social interaction and language change, perseverance, and depression (Table 1).

Of the studies evaluated, a small percentage of individuals, about 2.2 to 14%, presented side effects with the use of cannabis products, such as sleep disorders, restlessness, nervousness and change in appetite, in addition to moderate irritability, diarrhea, increased appetite, conjunctival hyperemia, behavioral problems, decreased cognition, fatigue and aggression/agitation.^16,17^ There was a psychotic symptom in one child, in a single-case study^17^; she interrupted treatment with CBD and THC and switched to ziprasidone 1.4 mg/kg/day. The symptoms resolved after 9 days.

## Discussion

Autism is part of a group of serious neurodevelopmental diseases that begin early in life and for which no specific treatment is available so far. ASDs are characterized by altered social interaction, compromised verbal and nonverbal communication, stereotyped and repetitive behaviors,^1^ often associated with social comorbidities^3,33,34^ and generalized anxiety.^35-37^

This systematic review sought to investigate whether cannabis-based products could bring any benefit to patients with ASD. From the nine studies evaluated, it was possible to observe that the cannabis products used were able to improve some symptoms related to ASD, e.g., self-mutilation and anger bouts, hyperactivity, sleep problems, anxiety, psychomotor agitation, irritability, aggressiveness, sensory sensitivity, cognition, attention, social interaction, language change, depression, and especially restlessness.

*Cannabis sativa* has over 500 identified active chemical constituents, and about 100 of them are classified as phytocannabinoids. The main phytocannabinoid is THC, responsible for the psychoactive effects of the plant, followed by CDB, exempt from this activity.^38-40^

The promising results found in this systematic review may be associated with the action of the phytocannabinoids present in the plant on the regulation of the endocannabinoid system. The endocannabinoid system is a unique biological system that affects a wide range of biological processes, including brain development and functioning. It consists of cannabinoid receptors (CB1 and CB2, mainly expressed in the brain and periphery, respectively), their endogenous ligands (endocannabinoids, mainly AEA and 2-arachidonoylglycerol [2-AG]), and enzymes for ligand synthesis and degradation.^19-22^ Endocannabinoids are key modulators of socioemotional responses, cognition, seizure susceptibility, nociception and neuronal plasticity,^23-26^ all of which are affected in ASD.

Endocannabinoids are known to regulate the main brain functions that are altered in ASDs.^41^ A well validated animal model of ASD based on prenatal exposure to valproic acid in rats has been used to evaluate behavioral alterations.^42,43^ There is strong evidence suggesting that altered levels of AEA, which already manifest in childhood and persist in adolescence and adulthood, may be associated with autistic symptoms, thus providing preclinical justification for a potential role of AEA signaling as a new therapeutic target for ASD. These results have corroborated a series of preclinical data that suggest that AEA signaling seems to play a modulating role on rodent behaviors associated with symptoms of ASD.^21^ A pioneering clinical study was able to identify low levels of AEA in plasma from children with ASD compared to plasma from children without ASD. These preliminary results corroborate the preclinical evidence that signs of AEA may be impaired in patients with ASD.^44^

A study conducted in 2019 by Aran et al.^25^ showed strong evidence that serum levels of certain endocannabinoids, mainly AEA and its structurally related compounds, are substantially reduced in people with ASD, regardless of age group or gender. That study has several limitations: uncontrolled retrospective study of a subgroup of children with severe and refractory behavioral problems; participants used several cannabis strains from different growers and a wide range of doses of CBD and THC; and the number of participants was not large enough to assess the impact of treatment on different subgroups of ASD.

It is important to note that endocannabinoids are not stored in any cell compartment for later use. They are generated on demand from the post-synaptic neuron cell membrane and are rapidly inactivated by pre-synaptic cell uptake and enzymatic hydrolysis. As a result, the concentrations of the various endocannabinoid pathways in the brain are constantly regulated, and even small changes in these concentrations can be clinically significant.^25^

Most of the studies evaluated in this systematic review used cannabis oil with higher CBD content when compared to the other phytocannabinoids present in the oil, at different proportions. Some studies have shown that CBD is capable of inhibiting the fatty acid amide hydrolase (FAAH), an enzyme responsible for the degradation of AEA, increasing its levels in the synaptic cleft^45,46^; this increase may be associated with an improvement in some ASD symptoms after use of CBD-rich cannabis products.

In a single-case study, Kurz & Blaas^27^ demonstrated that dronabinol, a synthetic analogue of THC, in doses ranging from 0.62 to 3.62 mg/day, was able to improve symptoms of hyperactivity, aggression, stereotyped and inappropriate speech. Notwithstanding, pure THC has not been commonly used, because the substance is responsible for most of the psychoactive effects of the plant.^47^ Crippa et al.^48^ report that CBD is capable of preventing the induction of psychotic symptoms induced by THC, suggesting that both substances could be useful used in combination.

Sometimes, patients with ASD need to make use of typical and atypical antipsychotics, anticonvulsants and mood stabilizers to control behavioral-mental changes such as psychomotor agitation and self- and/or heteroaggressiveness; psychostimulants and the antihypertensive clonidine to improve concentration and/or hyperactivity; serotonin inhibitors to improve obsessive-compulsive symptoms, anxiety disorders, depression and stereotypes. However, these drugs can cause serious side effects, such as nephropathy, hepatopathy, and metabolic syndrome, among others.^9^

In this systematic review, the cannabis products used in patients with ASD showed mild and moderate side effects, such as sleep disturbances, restlessness, moderate irritability, diarrhea, increased appetite, conjunctival hyperemia, behavioral problems, decreased cognition, fatigue, and aggression/agitation^16,17^ – effects not as severe as those observed with classic drugs.

In one of the studies included in this review, Adams et al.^30^ investigated the effectiveness of marijuana in a variety of diseases, including autism. Participants in this study used the plant, containing both CBD and THC, in different forms: flower, edible, vaporized, chewing gum, dye, leaf, oil, as well as isolated CBD. They observed improvements in some symptoms associated with ASD, e.g., anxiety, irritability, aggression, hyperactivity, and sleep, with mild adverse effects. Therefore, it is possible to observe that cannabis products appear to be safer when compared to the drugs traditionally used in the treatment of ASD-related symptoms.

Of the articles evaluated, only the three double-blind, placebo-controlled clinical trials that used CBD or CBDV alone assessed the influence of the substance on the central nervous system through functional magnetic resonance imaging (fMRI).^26,28,32^ Moreover, there was a difference in the results found in the images in relation to the placebo group, but the evolution of clinical symptoms or side effects could not be evaluated, as individuals used cannabis only once.

### fMRI pattern after using CBD

A 600 mg CBD oral solution was used in individuals with ASD who underwent fMRI to assess the effects of this treatment on their central nervous system.^26,28,32^ All those studies were carried out by the same team of researchers; 17 neurotypical adults and 17 adults with autism were administered a 600 mg CBD oral solution at one occasion, and a placebo substance at another occasion (randomized order); patients were then examined using fMRI. CBDV increased the levels of glutamate in the left basal ganglia, assessed with spectroscopy; however, in patients with ASD, despite the increase, the basal concentration of the substance decreased.^32^

It was noticed that CBD and CBDV altered the GABAergic system in all participants. The excitatory mechanisms of response to glutamate did not differ between the two groups, however the inhibitory response mediated by GABA was different in people with ASD, indicating that the brain of an autistic individual has a distinct GABAergic system from that of neurotypical individuals. In other words, the autistic brain reacts differently to GABA, and this discovery may help understand the mechanisms and targets of treatment in autism. Pretzsch et al.^28^ were pioneers for publishing the first evidence of neuromodulation made from the administration of CBD in fractional amplitude of low-frequency fluctuations and connectivity function in the brains of adults with ASD.

This finding is consistent with previous studies that pointed out differences in the functioning of the GABAergic system of people with autism and typical individuals, without the use of any substance.^49^ In addition, CBD was able to change the fractional low-frequency oscillation amplitude and functional connectivity in the adult brain in key regions commonly associated with the ASD condition. The authors of all studies did not mention any data about side effects or cognitive and/or behavioral changes. Also, it must be considered that the effects of a single administration were observed, and it is therefore not possible to predict long-term results of use.^28^

For Gallily et al.,^50^ the ideal form would be the use of the CBD-enriched extract; according to the authors, the use of isolated CBD brings a bell-shaped dose-response relationship, which would limit its clinical use. Conversely, the extract brought an increasing result after increasing the dose, improving anti-inflammatory and anti-nociceptive responses in mice.

### Clinical results of using cannabis to treat ASD symptoms without magnetic resonance imaging

The six articles that observed the effect of cannabis on the clinical aspects of children, adolescents and adults with ASD showed improvements in several behavioral aspects, regardless of the substance or composition employed. However, comparing the magnitude of the results is not possible, as the authors used different designs to measure and present the results – what they do have in common is the suggestion that cannabis could be a therapeutic alternative to autism. In all six articles evaluated, it was possible to observe an improvement in the following symptoms associated with autism: decreased bouts of self-mutilation and anger, hyperactivity, sleep problems, anxiety, restlessness, psychomotor agitation, irritability, perseverance, aggressiveness, and depression. Improvement in sensory sensitivity, cognition, attention, social interaction, and language were also reported. These results confirm the prediction of Khalil,^51^ who mentioned the need for systematic investigations into ASD and cannabis. That author argued that the tranquilizing, sedative and anticonvulsant properties of cannabis could assist in the main difficulties faced by children with autism, recognizing the behavioral and cognitive evolutions of cannabis in other pathologies and making a bridge with the mentioned results.

Most of the studies evaluated in this systematic review measured the evolution of symptoms through the perception of improvement by parents/caregivers of symptoms secondary to ASD, using questionnaires or scales developed by the authors themselves. None of the articles mentioned the use of neuropsychological assessments to investigate cognitive aspects.

Fleury-Teixeira et al.^31^ warned about the need for extra vigilance and a gradual increase in the dose of cannabinoids in patients using other psychotropic drugs. In those authors’ study, the symptoms of drowsiness, irritability, diarrhea, increased appetite, conjunctival hyperemia, and increased body temperature were seen in some cases and considered mild and/or transient. Few participants had to interrupt treatment before the end of the first month, due to adverse effects such as insomnia, irritability, rapid heartbeat, and worsening of the psychobehavioral crisis. The patients who had relevant side effects were all taking several medications, including at least one antipsychotic. A possible bias could be that the presence of epilepsy (38.9% of participants) may have interfered with the outcome, as studies that report improvement in epilepsy often also describe ASD-related symptoms.

It is important to highlight that all the randomized double-blind studies found on the use of cannabis and cannabinoids for autism assessed brain structures through magnetic resonance imaging, but did not have a focus on the efficacy and safety of cannabis for ASD. All evaluations were observational, either in individuals who started the medication in the study and were observed prospectively, or in those who had already used the substance and were analyzed retrospectively.

As general limitations of the studies included in this systematic review, it possible to cite the absence of follow-up evaluations and the lack of laboratory tests to help confirm the safety of the substances used. Also, only six studies evaluated the patients clinically; the others were based on image examination only. Samples were small, and several participants were lost along the study period. Finally, endocannabinoids were not dosed.

## Conclusion

Cannabis and cannabinoids have very promising effects in the treatment of autistic symptoms and can be used in the future as an important therapeutic alternative to relieve those symptoms, especially bouts of self-mutilation and anger, hyperactivity, sleep problems, anxiety, restlessness, psychomotor agitation, irritability, and aggressiveness; as well as improve sensory sensitivity, cognition, attention, social interaction, language, perseverance, and depression.

In addition, it is important to note that CBD can also change the levels of glutamate, glutamine and GABA, substances that contribute to the regulation of excitatory and inhibitory neurotransmission in both neurotypical and autistic individuals. However, randomized, double-blind and placebo-controlled clinical trials, as well as longitudinal studies, are necessary to clarify the findings on the effects of cannabis and its cannabinoids in individuals with autism.

Cannabis has been prescribed on an individual basis only, with autism being the second largest disease with available use, surpassed only by epilepsy. Therefore, it is essential to analyze what we have so far in the scientific literature, as cannabis is already being used worldwide as a phytopharmaceutical or as a CBD-rich cannabis extract for the autism spectrum.
